# Histological scoring of immune and stromal features in breast and axillary lymph nodes is prognostic for distant metastasis in lymph node‐positive breast cancers

**DOI:** 10.1002/cjp2.87

**Published:** 2018-01-08

**Authors:** Anita Grigoriadis, Patrycja Gazinska, Trupti Pai, Sheeba Irhsad, Yin Wu, Rosemary Millis, Kalnisha Naidoo, Julie Owen, Cheryl E Gillett, Andrew Tutt, Anthonius CC Coolen, Sarah E Pinder

**Affiliations:** ^1^ Breast Cancer Now Research Unit, Innovation Hub, Cancer Centre at Guy's Hospital, Faculty of Life Sciences and Medicine, King's College London London UK; ^2^ School of Cancer and Pharmaceutical Sciences CRUK King's Health Partners Centre, King's College London, Innovation Hub, Cancer Centre at Guy's Hospital, Great Maze Pond London UK; ^3^ Cancer Bioinformatics, King's College London, Innovation Hub, Cancer Centre at Guy's Hospital, Great Maze Pond London UK; ^4^ Department of Pathology Tata Memorial Centre, Tata Memorial Hospital Mumbai Maharashtra India; ^5^ Peter Gorer Department of Immunobiology King's College London Borough Wing, Guy's Hospital, London UK; ^6^ Breast Cancer Now, The Institute of Cancer Research London UK; ^7^ Institute for Mathematical and Molecular Biomedicine King's College London, Hodgkin Building, Guy's Campus London UK

**Keywords:** immune and stromal tumour‐environment, multivariate distant metastasis free survival analysis, lymph nodes, triple negative breast cancers, lymph node positive breast cancer

## Abstract

The prognostic importance of lymph node (LN) status and tumour‐infiltrating lymphocytes (TILs), is well established, particularly TILs in triple negative breast cancers (TNBCs). So far, few studies have interrogated changes in involved and uninvolved LNs and evaluated if their morphological patterns add valuable information for the prediction of disease progression in breast cancer. In a cohort of 309 patients enriched for TNBCs (170/309), we histologically characterised immune and stromal features in primary tumours and associated involved and uninvolved axillary LNs on routine haematoxylin and eosin stained sections. Of the 309 patients, 143 had LN‐positive disease. Twenty‐five histopathological features were assessed, including the degree of TIL presence, quantitative and qualitative assessment of germinal centres (GCs) and sinus histiocytosis. Multivariate and cross‐validated proportional hazard regression analyses were used to identify optimal covariate sets for prediction of distant metastasis‐free survival (DMFS). The degree of intratumoural and peritumoural immune infiltrate was associated with architectural changes in both uninvolved and involved LNs. By including clinicopathological characteristics as well as tumour and LN histopathological features in L2‐regularised proportional hazard models, the prediction of 5‐year DMFS was improved by 3–15% over the baseline in all cancers and in TNBCs. In LN‐positive cancers, the combination of Salgado's classification, lymphocytic lobulitis, size and number of GCs in the uninvolved LNs and location of GCs in the involved LNs carried significant prognostic information. From these features, a multivariate cross‐validation‐stable risk signature was constructed, which identified low‐risk groups within both LN‐positive breast cancers and the LN‐positive TNBCs group with a 10‐year DMFS probability of 78 and 87%, respectively. This study illustrates that, by incorporating histopathological patterns of involved and uninvolved LNs combined with primary tumour immune and stromal features, the prediction of developing distant metastasis in LN‐positive breast cancers can be estimated more accurately.

## Introduction

In invasive breast cancer, tumour size and the number of involved lymph nodes (LNs) have an inverse linear relationship with prognosis 1 and guide clinical decisions. Historical 10‐year survival after local therapy alone (surgery and radiotherapy) in patients with node negative disease is ∼85%, whilst for patients with involved axillary LNs it is 40–50% 2. Emerging data have identified patients with a low risk for recurrence amongst LN‐positive breast cancers who may be spared aggressive treatments. LNs are classified as involved with metastasis by pathological examination of conventional haematoxylin and eosin (H&E) sections, depending on the number of tumour cells and size of the largest malignant deposit 3. However, controversy regarding thresholds for these variables in addition to results from recent clinical trials in which occult metastasis were retrospectively evaluated 4, illustrate the uncertainty around the prognostic LN features for disease recurrence.

Accumulating evidence supports the prognostic and predictive role of the host immune response in early stage breast cancers, especially in oestrogen receptor (ER)‐negative, progesterone receptor (PR)‐negative, and HER2‐negative (i.e. triple negative) breast cancers (TNBCs) 5, 6, 7. Recent studies have shown that stromal tumour‐infiltrating lymphocytes (TILs), as opposed to intratumoural TILs, are potentially useful biomarkers in predicting response to therapy and overall outcome, when assessed via light microscopy of H&E stained tissue 5, 8, 9. Most studies to date have focused on the assessment of TILs within the primary tumour whilst largely ignoring peritumoural and nodal patterns of immune infiltrates. Clinical relevance of cell type specific alteration in LNs was provided by Kohrt *et al*, who demonstrated that the number of CD4 T cells and CD1a dendritic cells in axillary LNs allowed a more significant stratification of disease‐free survival for 77 breast cancer patients with small‐ and medium‐sized tumours than all other clinicopathological features 10.

The aims of this study were (i) to comprehensively catalogue the histopathological features of immune cell and other mesenchymal stromal infiltrates within tumour tissue, peritumoural tissue, and axillary nodal tissue and (ii) to determine whether any of these features are of prognostic value in breast cancer. Given the large number of features evaluated in relation to the small‐sized patient cohorts, we implemented an L2‐regularised multivariate Cox proportional hazard model with repeated cross‐validation to identify robust and generalisable putative predictors of developing distant metastasis.

## Materials and methods

### Study design and patients

This is a retrospective study of patients with invasive breast carcinoma treated between 1984 and 2002 at Guy's Hospital London, UK. Ethical clearance was obtained from the local research ethics committee. H&E stained sections of formalin‐fixed paraffin embedded tissue from primary invasive breast carcinomas, along with their involved and uninvolved LNs, were retrieved from the King's Health Partner's Breast Cancer Tissue and Data Bank (London, UK) from 309 patients. The cohort was enriched for patients with TNBC (*N* = 170, 142 of which were described previously 11), whilst 139 patients had non‐TNBC [hormone receptor‐positive/HER2‐negative (*N* = 62), hormone receptor‐negative/HER2‐positive (*N* = 59) and hormone receptor‐positive/HER2‐positive (*N* = 18)]. LN status was available for 276/309 patients. One hundred and forty‐three (52%) patients were LN‐positive whilst 133 (48%) had LN‐negative disease. Uninvolved axillary LNs were available for 134/143 patients with LN‐positive disease. In the remaining nine LN‐positive patients, all harvested nodes were involved. Clinicopathological data for each patient, including age at diagnosis, baseline tumour characteristics such as invasive size, histological grade, histological subtype, ER, PR, and HER2 status, and immunohistochemistry (IHC) for cytokeratins (CK) 5 and 14 and epidermal growth factor receptor (EGFR) status, LN involvement, and distant metastases were recorded (Table 1) and some have previously been described 11.

**Table 1:** Clinicopathological features of all breast cancers and TNBCs

Clinicopathological features	All breast cancers *N*=309 (%)	TNBC *N*=170 (%)
*Age at diagnosis*		
Below 50 years	106 (34)	61 (36)
Over 50 years	203 (66)	109 (64)
*Tumour size (cm)*		
pT1	68 (22)	35 (21)
pT2	183 (59)	103 (61)
pT3	45 (15)	26 (15)
Unknown	13 (4)	7 (4)
*Histological grade*		
I	9 (3)	1 (0.5)
II	47 (15)	7 (4)
III	253 (82)	162 (95.5)
*Histological subtypes*		
Invasive breast carcinoma of no special type	253 (81)	143 (84)
Mixed ductal and lobular carcinoma	24 (8)	10 (5.8)
Invasive lobular carcinoma	11 (4)	5 (2.9)
Carcinoma with apocrine differentiation	9 (3)	2 (1.2)
Metaplastic carcinoma of no special type	4 (1)	4 (2.4)
Invasive papillary carcinoma	2 (0.6)	2 (1.2)
Carcinoma with medullary features	2 (0.6)	2 (1.2)
Salivary gland/skin adnexal type tumors	2 (0.6)	2 (1.2)
Adenosquamous carcinoma	1 (0.3)	0
Secretory carcinoma	1 (0.3)	0
*DCIS*		
Present	143 (46)	66 (39)
Absent	163 (53)	102 (60)
Unknown	3 (1)	2 (1)
*Necrosis*		
Present	106 (34)	61 (36)
Absent	203 (66)	109 (64)
*Fibrosis*		
Present	90 (29)	54 (36)
Absent	216 (70)	115 (64)
Unknown	3 (1)	1 (0.5)
*Lymphovascular invasion*		
Present	90 (29)	45 (26)
Absent	216 (70)	124 (73)
Unknown	3 (1)	1 (0.5)
*Lymph node status*		
Positive	143 (46)	64 (38)
Negative	133 (43)	81 (47)
Unknown	33 (11)	25 (15)
*Distant metastasis*		
Present	129 (42)	59 (35)
Absent	180 (58)	111 (65)
*Immunohistochemical subtypes*		
Hormone receptor+/HER2–	62 (20)	NA
Hormone receptor–/HER2+	59 (19)	NA
Hormone receptor+/HER2+	18 (6)	NA
Triple Negative	170 (55)	170 (100)
*EGFR*		
Present	67 (22)	53 (31)
Absent	225 (73)	106 (63)
Unknown	17 (5)	11 (7)
*CK5/6*		
Present	68 (22)	57 (3)
Absent	225 (73)	103 (61)
Unknown	16 (5)	10 (6)
*CK14*		
Present	49 (16)	44 (26)
Absent	244 (79)	114 (67)
Unknown	16 (5)	12 (7)

### Histopathological immune assessment

#### Classification of immune infiltrates

All routine H&E stained sections of primary breast tumours and uninvolved and involved LNs were reassessed histopathologically. A minimum of 6 sections of the primary tumour and 1–12 (mean = 6) LNs per case were available for evaluation of immune and stromal features. All sections were reviewed by a specialist Consultant Breast Pathologist (SEP) and Pathology Research Scientist (PG). Analyses of the tumour microenvironment were performed using conventional microscopy of H&E whole sections. The NanoZoomer HT Digital Pathology Scanning System (Hamamatsu, Japan), was used to scan H&E sections with a spatial resolution of 0.46 μm/pixel for the measurement of LN germinal centres (GCs). According to recommendations by the International Tumour Infiltrating Lymphocytes Working Group 12, morphological features initially assessed as continuous variables (e.g. stromal TILs) were discretised for statistical analysis. All features and their associated categories described below in detail are summarised in supplementary material, Tables S1A and S1B.

#### Intratumoural site

**Figure 1:**
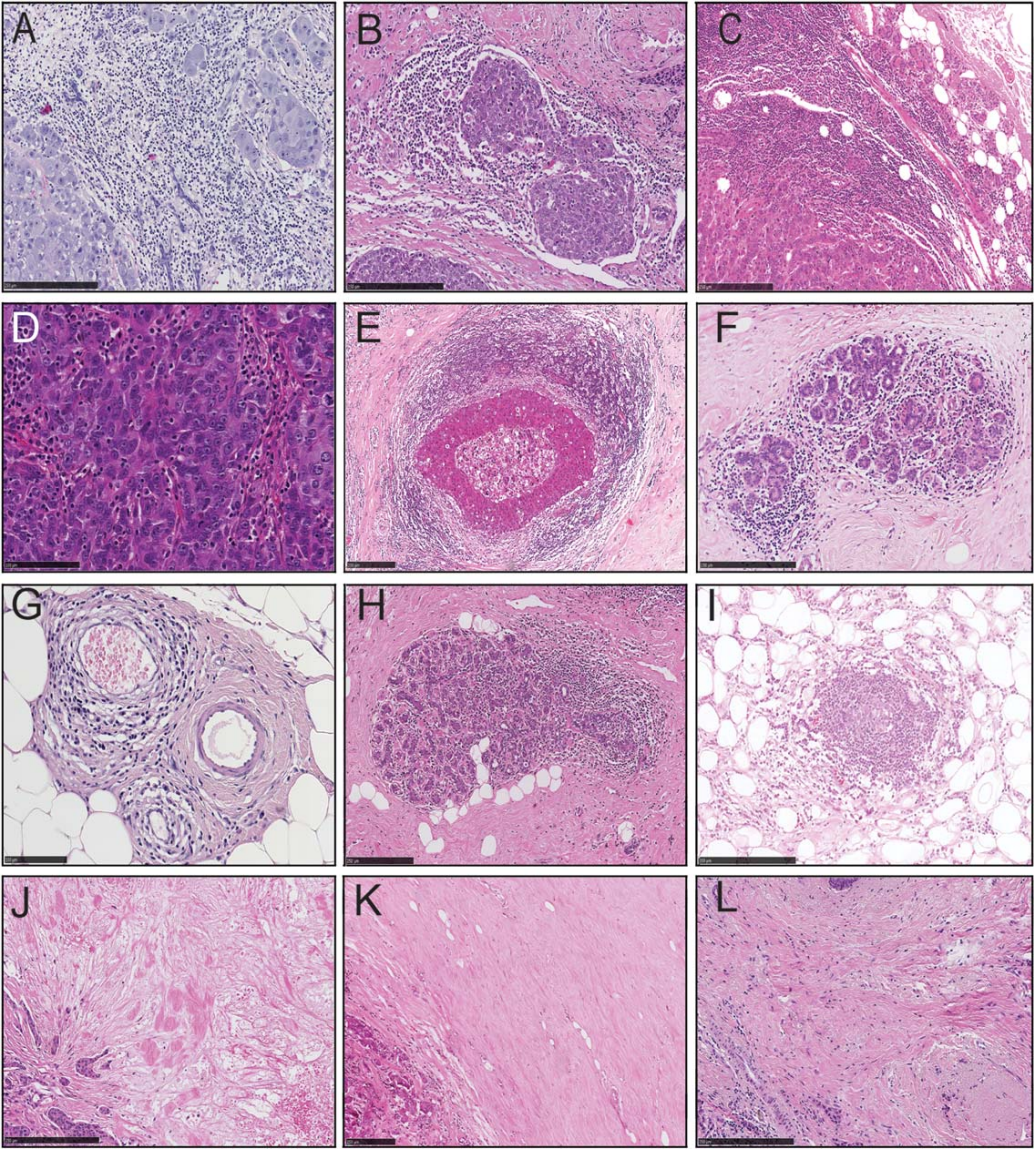
Morphological assessment of the immune parameters in the intra‐tumoural and peri‐tumoural sites of the primary tumour (H&E stain). (i) Intratumoural site: (A) TILs scattered in the intratumoural stroma (score 4; ≥50%; strong); (B) TILs around tumour cell nests, (score 4; ≥50%; strong); (C) TILs at the invasive tumour margin (score 4; ≥50%; strong); (D) presence of intratumoural TILs (lymphocytes present within tumour cell nests ‘intra‐epithelial’). (ii) Peri‐tumoural components: (E) lymphocytic infiltrate surrounding DCIS (score 3; ≥50%; strong); (F) lymphocytic infiltrate surrounding normal breast lobules (score 3; ≥50%; strong); (G) perivascular lymphocytic infiltrate (score 3; ≥50%; strong); (H) presence of lymphocytic lobulitis; (I) presence of TLS (lymphoid GC formation). Stromal features in the tumour environment: (J) oedematous/myxoid; (K) hyalinised stroma; (L) fibroblastic stroma.

Immune infiltrates were assessed semi‐quantitatively across the entire tumour section and then in different tumour regions. As per Salgado's criteria, TILs were classified as ‘stromal TILs’ (Figure 1A–C) or ‘intratumoural TILs’ (Figure 1D). Stromal TILs were further sub‐classified as TILs scattered within the tumour stroma (Figure 1A); TILs around tumour cell nests (Figure 1B) and TILs seen at the invasive margin (Figure 1C). The degree of TILs present was further graded semi‐quantitatively as: 0 = absence of lymphocytes, 1 = minimal (1–10% of surface area in a given location); 2 = mild (10–20%); 3 = moderate (>20–50%); and 4 = strong (≥50%).

#### Peritumoural site (including premalignant and non‐tumorous components)

Immune infiltrates associated with *in situ* and normal tissue adjacent to invasive tumours were assessed when seen (i) next to DCIS (Figure 1E), (ii) in normal breast lobules (Figure 1F), and (iii) in perivascular areas (Figure 1G). Lymphocytic lobulitis 13 (Figure 1H) and tertiary lymphoid structures (TLS) were graded as present or absent (Figure 1I).

#### Stromal features

Assessment of stromal features in the invasive tumours was based on their overall appearances and recorded as oedematous/myxoid (Figure 1J), hyalinised (Figure 1K) and/or fibroblastic (Figure 1L); when a mixed pattern was present, the predominant feature was recorded. Smooth muscle actin and alcian blue staining of selected cases supported the H&E stromal classification (supplementary material, Figure S1).

#### Involved and uninvolved axillary LNs

Three main morphological immune features were assessed in axillary involved LNs: (A) GC features, (B) degree of sinus histiocytosis, and (C) pattern of metastatic tumour involvement (supplementary material, Table S1B). In uninvolved LN, only (A) and (B) were assessed.

**Figure 2:**
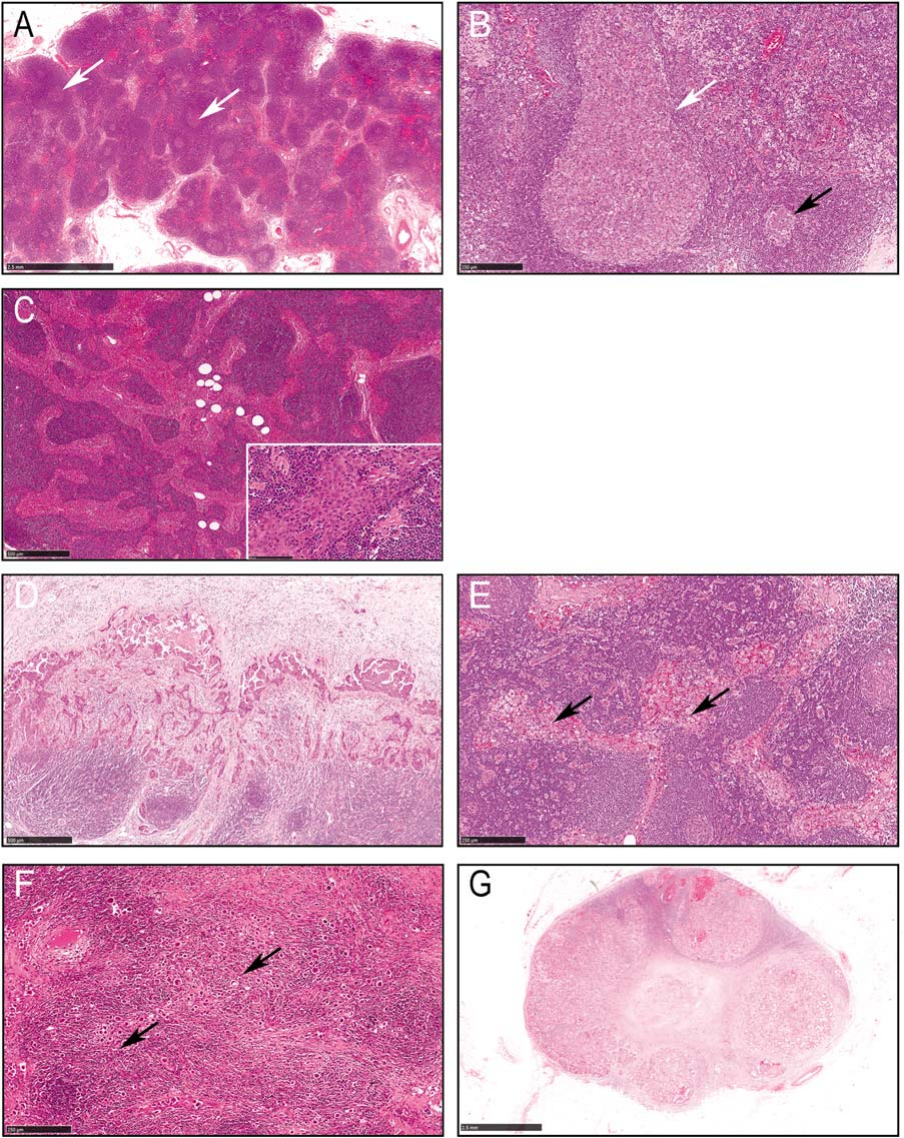
Histomorphological features of uninvolved and involved LNs (H&E stain). In the uninvolved LNs: (A) numerous GCs (grade 3) GC located throughout the LN (white arrows); (B) large GC (white arrow) with an adjacent small GC (black arrow); (C) sinus histiocytosis grade 4 (inset showing a higher power view). In the involved LNs: (D) sub‐capsular metastasis; (E) sinusoidal pattern (black arrows); (F) diffuse pattern (black arrows); (G) nodular pattern with near total replacement of the nodal tissue with metastatic deposits.

(i) The number of GCs was categorised as grade 0 (absent), 1 (few), 2 (moderate), or 3 (numerous, frequently distributed throughout the LN) (Figure 2A). (ii) The distribution of GCs was classified as predominantly peripheral (majority close to the capsule), predominantly central (most GCs in the centre of the LN) (Figure 2A) or mixed architecture (GCs were located across the whole LN). (iii) Average size of GCs was classified as either small (<200 µm in diameter), moderate (200–400 µm), large (>400 µm), or mixed (if more than one size) (Figure 2B). In those with mixed sizes of GCs, cases with a predominant pattern of large GCs were further classified as ‘GC hyperplasia’. Supplementary material, Figure S2 shows staining with CD20 (B cell marker) and CD11c (dendritic cell marker) on selected cases, which corroborated the classification of morphological changes in the GCs of the LNs.

Sinus histiocytosis was assessed using a modification of the method previously described by Culter *et al*, 14. The number of histiocytes across the sinuses was classified as: grade 0 (absent), grade 1 (<2 cells), grade 2 (2–4 cells), grade 3 (>4 to <8 cells), or grade 4 (≥8 cells across the sinus) (Figure 2C). The dilatation of sinuses by cells other than histiocytes, for example, by metastatic tumour cells, or other inflammatory cells or oedema fluid, was ignored.

Metastatic tumour spread within the LN was classified into six patterns: (i) sub‐capsular (within the sinus immediately under the LN capsule) (Figure 2D), (ii) intra‐sinusoidal [within sinuses in the body of the LN (Figure 2E)], (iii) diffuse (Figure 2F), (iv) nodular (Figure 2G), (v) mixed (mixture of several patterns), and (vi) total replacement by tumour.

### Data analysis and interpretation

**Figure 3:**
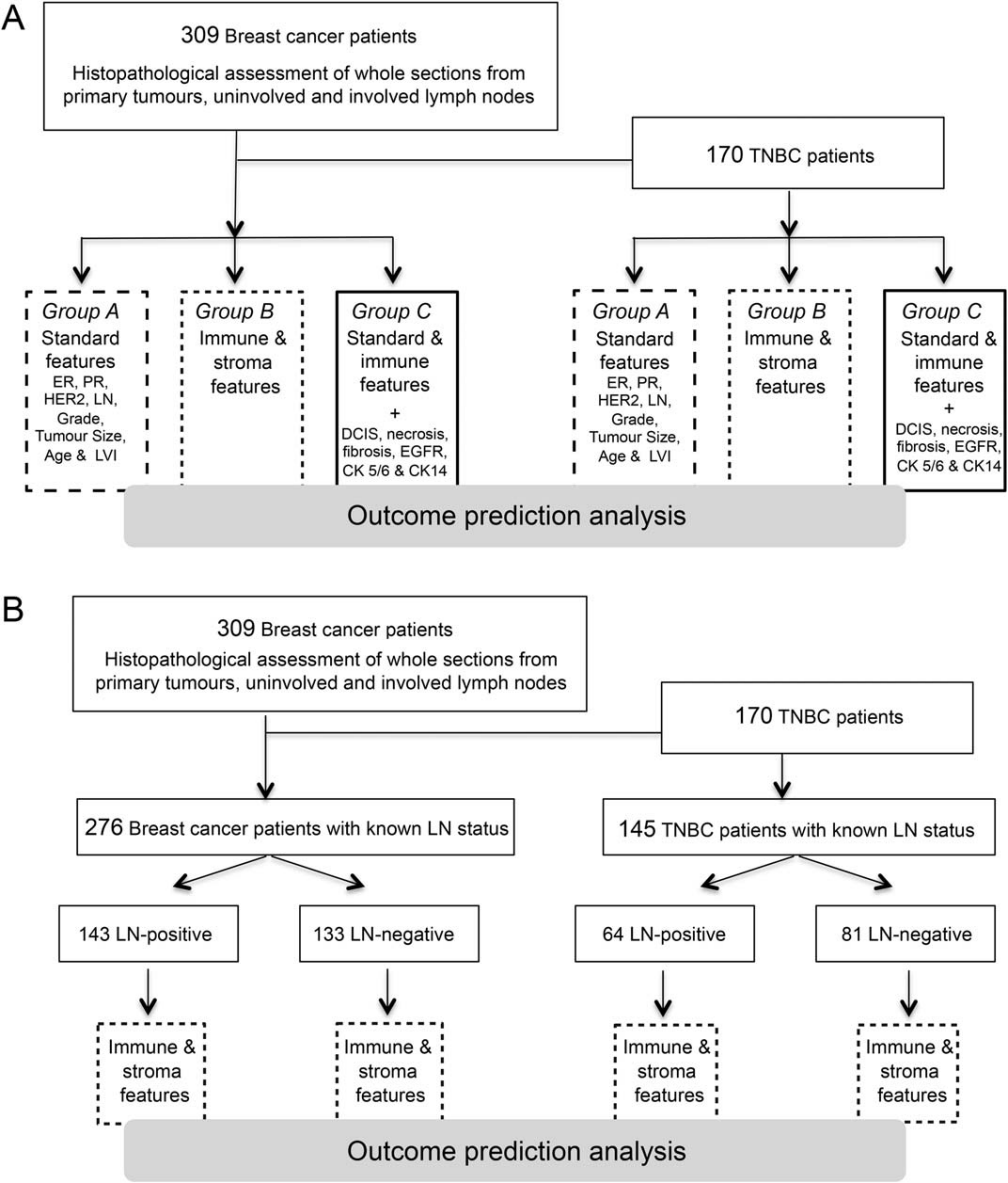
Consort diagram of cohorts used in the optimised multivariate proportional hazard model. (A) 309 breast cancer and 170 TNBC patients were analysed with group A (standard features, big dashed box); group B (immune & stroma features, small dashed box) and group C (combination of standard and immune& stroma, plus additional 8 characteristics, black box). (B) Stratification of cohorts into LN‐positive and LN‐negative patients. Multivariate proportional hazard model was applied using group B features (immune & stromal features, small dashed box).

The end point of the study was distant metastasis‐free survival (DMFS), which was calculated from the date of diagnosis of the primary tumour to the first event of any distant metastases. An L2‐regularised multivariate proportional hazard model, based on iterative determination of optimal covariates to prevent overfitting via repeated cross‐validation, was applied to all breast cancers (*N* = 309), TNBC (*N* = 170) patients, and their LN status dichotomised subgroups 15, 16, 17, 18. Three sets of covariates were used in the analytical models: group A included eight clinicopathological features, group B encompassed histologically assessed immune and stromal features and group C was a combination of A and B. Figure 3 provides a CONSORT diagram explaining cohorts used for statistical analyses. A detailed description of the statistical analysis is provided in supplementary material, Supplementary materials and methods and Figure S3. In brief, each analysis was performed with 100 iterations and their results were averaged. The fraction of correctly predicted patients was recorded after 1 year, and every year up to 10 years of DMFS. At each time point, an optimal set of covariates with the highest prediction accuracy was determined by ranking the covariates according to their relevance in the cross‐validated Cox regression analysis. Regression parameters for each covariate were reported as hazard ratios (HRs) with 95% confidence intervals (CI) 19.

For LN‐positive cohorts, an optimal covariate‐set to predict 5‐year DMFS was established to construct a single‐patient immune and stromal histopathological (ISH) risk score, by summing the standardised covariates weighted by their coefficients. LN‐positive patients were grouped into those with low and upper quartile, and mean risk signature scores. Kaplan‐Meier estimators were used, and the log‐rank test was performed to test differences among groups.

## Results

### Clinicopathological characteristics of the patients

The clinicopathological features of the 309 evaluated patients are presented in Table 1. First analysis was restricted to TNBC patients (*N* = 170; 81 with LN‐negative and 64 with LN‐positive disease and 25 patients within unknown LN status), followed by randomly selected non‐TNBC patients (*N* = 139; 52 with LN‐negative, 79 LN‐positive, and 8 with unknown LN status). All patients were female with a median age of 55 years (range = 24–89) at diagnosis. Most (*N* = 183; 59%) patients had T2 tumour size and a predominant number of cases (*N* = 253; 82%) were histologically grade 3, as might be expected in a cohort enriched for TNBC and for LN positive cases. Most cases were invasive breast carcinomas of no special type (82%). Lymphovascular invasion (LVI) was seen in 90 (29%) cases. Median follow‐up was 7.9 years for the entire cohort (range = 0.3–25). Distant metastasis was recorded in 129 (42%) patients, of whom 70% developed distant metastasis within the first 2.5 years after diagnosis (range of DMFS 0.3–18 years).

### Lymphocytic infiltration at invasive and peritumoural sites

#### Intratumoural sites

An absence of lymphocytic infiltration across the entire tumour was noted in 6 (3.5%) TNBC patients compared with 18 (12.9%) non‐TNBC patients. The majority of TNBC patients had a moderate (*N* = 78, 45.9%) or strong (*N* = 23, 13.5%) degree of lymphocytic infiltration across the entire tumour. In contrast, a majority of non‐TNBC patients had minimal (*N* = 45, 32.4%) or mild (*N* = 36, 25.9%) degree of infiltration. A strong degree of lymphocytic infiltration across the entire tumour was seen only in 5 (3.6%) non‐TNBC patients. Intra‐tumoral TILs were present in more TNBC patients (*N* = 51, 30%) compared with non‐TNBC patients (*N* = 10, 7.2%). Likewise, a strong degree of TILs at the invasive margins was noted in more TNBC (*N* = 22, 12.9%) than non‐TNBC patients (*N* = 2, 1.4%) (see Table 2A).

#### Peritumoural sites

In 130 of the total 309 cases, DCIS was seen in close proximity to the invasive tumour. In 48/130 (36.9%) cases, the DCIS was free of lymphoid infiltrate, whilst the rest had varying degrees of immune infiltrates. A strong lymphoid infiltrate was found surrounding breast lobules in the TNBC group (*N* = 40, 30.5%) compared with the non‐TNBC group (*N* = 20, 17.5%). Lymphocytic lobulitis and TLS in areas surrounding the invasive tumours were seen more frequently in TNBC cases (16 and 27.6%, respectively) compared with non‐TNBCs (9.6% and 17.3%, respectively) (see Table 2A).

#### Tumour stroma

Fibroblastic stroma was the most common intratumoural stromal change noted accounting for 87% cases, followed by hyalinised stroma in 86% cases and myxoid change was the least common noted in 23% cases. No differences were noted across tumour subgroups (Table 2B).

**Table 2:** Immune and stromal features in tumours and LNs: (A). Distribution of the immune features at tumoural and peri‐tumoural sites assessed across TNBC and non‐TNBC subgroups. (B). Distribution of the stromal features in TNBC and non‐TNBC subgroups. (C): Pattern of histomorphological features in involved and uninvolved lymph nodes in LN‐positive and LN‐negative patients

	TNBC	Non‐TNBC	
Characteristics assessed	*N* = 170 (%)	*N* = 139 (%)	*P* value
**A. Intratumoural and peritumoural assessment**			
*Semi‐quantitative assessment of lymphocytic infiltration across entire tumour*			
Absence of lymphocytes	6 (3.5)	18 (12.9)	
Minimal (1 to <10%)	18 (10.6)	45 (32.4)	
Mild (10–20%)	45 (26.5)	36 (25.9)	
Moderate (>20 to <50%)	78 (45.9)	35 (25.2)	Chi‐square test
Strong (≥50%)	23 (13.5)	5 (3.6)	6.94E‐09
*TILs scattered in the intratumoural stroma*			
Absence of lymphocytes	14 (8.2)	32 (23)	
Minimal (1 to <10%)	43 (25.3)	60 (43.2)	
Mild (10–20%)	50 (29.4)	24 (17.3)	
Moderate (>20 to <50%)	55 (32.4)	22 (15.8)	Fisher's exact test
Strong (≥50%)	8 (4.7)	1 (0.7)	1.86E‐07
*TILs around tumour cell nests*			
Absence of lymphocytes	41 (24.1)	61 (43.9)	
Minimal (1 to <10%)	33 (19.4)	35 (25.2)	
Mild (10–20%)	29 (17.1)	14 (10.1)	
Moderate (>20 to <50%)	55 (32.4)	24 (17.3)	Chi‐square test
Strong (≥50%)	12 (7.1)	5 (3.6)	2.68E‐04
*TILs at the invasive margin*			
Absence of lymphocytes	12 (7.1)	40 (28.8)	
Minimal (1 to <10%)	21 (12.4)	33 (23.7)	
Mild (10–20%)	44 (25.9)	33 (23.7)	
Moderate (>20 to <50%)	71 (41.8)	31 (22.3)	Chi‐square test
Strong (≥50%)	22 (12.9)	2 (1.4)	5.70E‐10
*Intratumoural TILs*			
Absent	119 (70)	129 (92.8)	Chi‐square test
Present	51 (30)	10 (7.2)	1.14E‐06
*Lymphocytic infiltrate surrounding DCIS*			
DCIS absent	108 (63.5)	71 (51.1)	
Absent	21 (33.9)	27 (39.7)	
Mild (10–20%)	11 (17.7)	17 (25)	
Moderate (>20 to <50%)	14 (22.6)	8 (11.8)	Chi‐square test
Strong (≥50%)	16 (25.8)	16 (23.5)	8.14E‐02
*Salgado's classification*			
0–10% stromal TILs	39 (22.9)	77 (55.4)	
20–40% stromal TILs	61 (35.9)	37 (26.6)	Chi‐square test
50–90% stromal TILs	70 (41.2)	25 (17.9)	9.70E‐09
*TILs surrounding normal breast lobules*			
No normal breast lobules	39 (22.9)	25 (17.9)	
Normal breast lobules			
Absent	26 (19.8)	35 (30.7)	
Mild (10–20%)	30 (22.9)	34 (29.8)	
Moderate (>20 to <50%)	35 (26.7)	25 (21.9)	Chi‐square test
Strong (≥50%)	40 (30.5)	20 (17.5)	3.25E‐02
*Perivascular infiltrate*			
Absent	28 (16.5)	22 (15.8)	
Mild (10–20%)	52 (30.6)	50 (35.9)	
Moderate (>20 to <50%)	53 (31.2)	37 (26.6)	Chi‐square test
Strong (≥50%)	37 (21.8)	30 (21.6)	7.44E‐01
*Lymphocytic lobulitis*			
No normal breast lobules	39 (22.9)	25 (17.9)	
Absent	110 (83)	103 (90.4)	Chi‐square test
Present	21 (16)	11 (9.6)	1.88E‐01
*Tertiary lymphoid structures*			
Absent	123 (72.4)	115 (82.7)	Chi‐square test
Present	47 (27.6)	24 (17.3)	4.32E‐02
**B. Stromal features**			
Oedemamtous/Myxoid stroma			
Absent	125 (73.5)	114 (82)	
Present	41 (24.1)	25 (18)	Fisher's exact test
Dominant	4 (2.4)	0 (0)	6.79E‐02
Hyalinised stroma			
Absent	21 (12.4)	21 (15.1)	
Present	129 (75.9)	103 (74.1)	Chi‐square test
Dominant	20 (11.8)	15 (10.8)	7.70E‐01
Fibroblastic stroma			
Absent	20 (11.8)	20 (14.4)	
Present	137 (80.6)	110 (79.1)	Chi‐square test
Dominant	13 (7.6)	9 (6.5)	7.50E‐01

	LN‐positive patients		LN‐negative patients
	Involved LN	Uninvolved LN	Uninvolved LN
Characteristics assessed	*N* = 143 (%)	*N* = 143 (%)	*N* = 133 (%)
**C. Lymph node**			
*GC assessable*	97 (67.8)	88 (61.5)	105 (78.9)
Complete absence of GC	26 (18.2)	46 (32.2)	28 (21.1)
Total metastatic replacement of LN	20 (14)	NA	NA
No uninvolved LN available for assessment	NA	9 (6.3)	NA
*GC semi‐quantitative assessment*			
Grade 1 (Few)	37 (38.1)	34 (38.6)	45 (42.9)
Grade 2 (Moderate)	41 (42.3)	31 (35.2)	29 (27.6)
Grade 3 (Numerous)	19 (19.6)	23 (26.1)	31 (29.5)
*GC location*			
Peripheral	51 (52.6)	34 (38.6)	50 (47.6)
Predominantly peripheral	19 (19.6)	27 (30.7)	23 (21.9)
Central	6 (6.2)	0 (0)	0 (0)
Predominantly central	21 (21.6)	27 (30.7)	32 (30.5)
*GC size*			
Small	23 (23.7)	20 (22.7)	32 (30.5)
Moderate	13 (13.4)	11 (12.5)	14 (13.3)
Large	4 (4.1)	4 (4.5)	4 (3.8)
Mixed	57 (58.8)	53 (60.2)	55 (52.4)
*GC hyperplasia*			
Absent	52 (53.6)	48 (54.5)	65 (61.9)
Present	45 (46.4)	40 (45.5)	40 (38.1)
*Sinus histocytosis*			
Grade 1 = <2 cells across	1 (1.1)	3 (2.2)	3 (2.3)
Grade 2 = 2–4	9 (9.5)	24 (17.9)	14 (10.5)
Grade 3 = >4 to <8	41 (43.2)	50 (37.3)	61 (45.9)
Grade 4 = ≥8	44 (46.3)	57 (42.5)	55 (41.4)
No uninvolved LN available for assessment	NA	9	NA
SH Mets replacement	48	NA	NA
*Metastatic pattern*			
Sub‐capsular	4 (2.8)	NA	NA
Sinusoidal	7 (4.9)	NA	NA
Diffuse	6 (4.2)	NA	NA
Nodular	45 (31.5)	NA	NA
Mixed	61 (42.7)	NA	NA
Total metastatic replacement of LN	20 (14)	NA	NA

#### Histological evaluation of involved and uninvolved axillary LNs

Primary tumours may lead to reactive and structural changes in regional LNs prior to development of nodal metastases 20, 21. A total of 267 uninvolved and 143 involved LNs were reviewed. GCs were present in 193 uninvolved LNs (72%) and 97 involved LNs (67%) and assessed for their number, location, and size. Within involved LNs, GCs were located mainly in the periphery (52.6%) or predominantly in the centre of LNs (21.6%) (Table 2C). The numbers of large GCs and the overall size distribution in uninvolved and involved LN was comparable (Table 2C). Similarly, the frequency of grades 3 and 4 sinus histiocytosis was comparable.

### Correlation of histopathological features of intratumoural, peritumoural, stromal, and nodal sites

We next investigated whether the degree and presence of these histological features occurred independently. Using a Pearson's correlation, there was a concurrent increase in the degree of lymphocytic infiltrate at the invasive tumour, around tumour nests, at the peripheral tumour edge and within the stroma (supplementary material, Figure S4). We also noticed that, in all breast cancers and particularly among TNBCs, the number and size of the GCs in uninvolved and involved LNs grew with increasing levels of immune infiltrates at the primary tumour site (*r*: 0.46, *p* <0.001; supplementary material, Figure S4).

### Immune‐associated characteristics improve prognostic accuracy for DMFS

**Figure 4:**
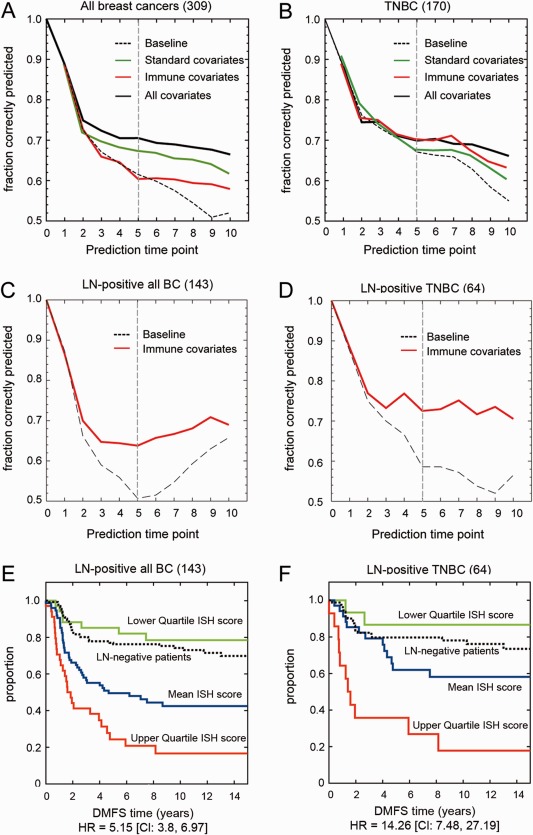
Optimised proportional hazards models to identify covariates for the prediction of developing distant metastasis in all invasive breast cancers and TNBC cohorts. Three different initial sets of covariates were used for the prediction analysis, namely 8 standard features (group A); 25 immune and stroma features (group B); and all available features in (group C). The dotted line in all graphs indicates the baseline performance without any covariates (i.e. based solely on any imbalance between cases and controls). The green, red, and black lines show the performance on the validation set including either groups A, B, and C, respectively. LN‐positive patients of all breast cancers (C) and TNBC (D) were analysed with group B covariates. The LN‐positive cohorts were further dichotomised based on the ISH‐risk score. Kaplan‐Meier curves for all breast cancers (E) and TNBC (F) illustrating the duration of DMFS according to lower quartile (green line), mean (blue line), and upper quartile (red line) of ISH‐risk score grouping. HR and CI are listed below the graph. The black dotted lines display the survival curves for LN‐negative patients of all breast cancers (*n* = 133) and TNBC (*n* = 81).

Next, we asked whether these novel histomorphological features carry prognostic information for the development of distant metastases. Given that several of these immune and stromal cell patterns do not occur independently (supplementary material, Figure S4), we used a Bayesian multivariate survival analysis algorithm optimised to undo the possible effects of overfitting 18. We analysed three groups of covariates (A, B, C) first in all breast cancers and then in the TNBC cohort only. We then evaluated their performance in correctly predicting the fraction of patients free of distant metastasis at 5 years after diagnosis in comparison to the baseline performance in which no covariates were used and in which the risk of distant metastasis is derived solely from any imbalance between cases and controls (Figure 4, vertical grey lines). Covariate‐group A included eight clinicopathological features; ER, PR, and HER2 status, histological grade, LN status, tumour size, age at diagnosis, and the presence or absence of LVI. Group B encompassed the 25 histologically assessed features described above in detail (14 features are from the primary tumour site and 11 features were obtained from involved and uninvolved LNs, supplementary material, Table S1). In group C, we combined groups A and B. In all breast cancers, covariate‐group A correctly predicted 5‐year DMFS in 68% of patients (green line, Figure 4A). This was a modest improvement compared to 62% baseline performance (dotted line, Figure 4A) and the covariate‐group B (red line, Figure 4A). The combined covariate‐group C performed best by correctly predicting 5‐year DMFS in 71% of patients (black line, Figure 4A). In TNBC, the differences in predictive accuracy between groups A, B, and C were less pronounced. Of note, within the TNBC cohort, covariate‐group B (red line, Figure 4B) was more accurate in predicting 5‐year DMFS (71%) than either group A (67%, green line, Figure 4B) or the baseline performance (67%, dotted line, Figure 4B). The optimal covariates among the covariate‐group B were selected as being risk‐predictive at 5‐year DMFS, namely TILs in the primary tumour according to Salgado's criteria 12, the number and size of GCs in the uninvolved LN, the location of GCs, and a diffuse metastatic pattern in the involved LN, as well as the presence of lymphocytic lobulitis in adjacent normal breast tissue (Table 3). Supplementary material, Table S2A provides the full list of optimal covariates selected at 5‐year DMFS in both cohorts for each of the three covariate‐groups used in these analyses.

**Table 3:** Immune, stromal and LN feature selection from multivariate Bayesian Cox regression analyses

	LN‐positive and LN‐negative						LN‐positive		LN‐negative	
	All breast cancers			TNBC			All breast cancers	TNBC	All breast cancers	TNBC
	Group A	Group B	Group C	Group A	Group B	Group C	Group B	Group B	Group B	Group B
Selected features to predict risk for developing distant metastasis	Standard	Immune and stroma	All features	Standard	Immune and stroma	All features	Immune and stroma	Immune and stroma	Immune and stroma	Immune and stroma
LN status	1.904		2.248	1.558		1.635				
ER status			0.7							
HER2 status	1.797		1.76							
LVI status	1.483			1.642						
Fibrosis			1.55							
Salgado's classification		0.67	0.629				0.687	0.282		
TILs at the invasive margin			0.509			0.571	0.487			
Lymyphocytic lobulitis					0.368	0.436	0.591	0.339		0.196
Lymphoid infiltrate surrounding DCIS						0.358				
Tertiary lymphoid structures						2.298				2.24
Oedematous/Myxoid stroma						1.889			2.22	3.32
GC, semi‐quantitative assessment in uninvolved LN		0.348	0.248		0.413	0.451	0.212	0.11		
GC, semi‐quantitative assessment in involved LN		0.541	0.654			0.496				0.426
GC, size in uninvolved LN		2.216	2.071				2.659	2.713		
GC, size in involved LN						3.504				
GC, location in involved LN		2.932	3.04		3.401	3.758	2.234	2.898		
GC, hyperplasia in uninvolved LN			1.59							
GC, hyperplasia in involved LN					0.371	0.278		0.666		
Metastatic pattern in involved LN					2.221	4.11		3.334		

Features are listed and their HR shown if it was selected from Group A (standard features), Group B (immune & stromal features), and Group C (all features). All cohorts that were used for analyses are reported. Covariates used for the Immuno‐stroma‐histological (ISH)‐risk score are indicated in grey.

### A risk score based on histomorphological features identifies LN‐positive breast cancers patients with low risk for distant metastasis

As morphological patterns in involved and uninvolved LNs carried prognostic value, we asked if these features were predictive for developing distant metastasis in both LN‐positive and LN‐negative disease. All breast cancers and TNBCs group were further divided into patients with LN‐positive (143 all cancers and 64 TNBC), and LN‐negative disease (133 all cancers and 81 TNBC). These four sub‐cohorts were then analysed independently with L2‐regularised proportional hazards models using covariate‐group B. In the LN‐positive cohorts, covariates were selected via cross‐validation for predicting a 5‐year DMFS, including Salgado's classification, the presence of lymphocytic lobulitis, the size, and number of GCs in the uninvolved LN and location of GCs in the involved LN (Table 3, supplementary material, Tables S2B and S2C). Incorporating these five features improved the 5‐year DMFS predictive accuracy from 50% baseline performance (dotted line, Figure 4C) to 64% in all breast cancers (red line, Figure 4C) and from 58% baseline performance (dotted line, Figure 4D) to 74% in TNBC (red line, Figure 4D). Amongst the two LN‐negative cohorts, covariate‐group B failed to improve 5‐year DMFS predictive accuracy (supplementary material, Figure S5). Due to the smaller datasets in these sub‐cohorts, residual overfitting cannot be excluded.

Lastly, we built an ‘immune‐stroma‐histological (ISH)‐risk score’ with these five histomorphological features (see supplementary material, Supplementary materials and methods), and grouped patients according to their ISH‐risk score levels. Patients with the lowest quartile ISH scores had a 10‐year DMFS of 78% (all breast cancer cohort) and 87% (TNBC cohort) compared with 17% (all breast cancer cohort) and 18% (TNBC cohort) for patients with an upper quartile ISH score (Kaplan‐Meier survival estimates; log‐rank test of difference in survival, *p* < 0.001, HR = 5.15, 95% CI = 3.8–6.97; *p* < 0.001, HR = 14.26, 95% CI = 7.48–27.19) (Figure 4E and 4F). Moreover, LN‐positive breast cancer patients with low ISH score had even less risk of developing distant metastases than LN‐negative breast cancer patients (Figure 4E,F, black dotted line). This suggests that histomorphological changes within uninvolved and involved LN carry significant prognostic information for the risk of distant metastasis amongst LN‐positive patients, even among TNBCs.

## Discussion

It is well known that routinely assessable clinicopathological characteristics such as LN status or the presence of TILs predict the risk of recurrence, distant metastases and overall outcome in breast cancer patients. In this study, we have utilised an optimised multivariate proportional hazard model for analysing ‘time‐to‐event’ data which was based on extensive H&E histopathological data to determine whether these features are of prognostic value in breast cancer. Reassuringly, well‐established prognostic markers were shown to be associated with outcome, including the presence of LN metastases whilst we also confirm the value of the Salgado classification 12. We report, for the first time, that the number and architecture of GCs in involved and uninvolved LNs, as well as the presence of lymphocytic lobulitis, provide important information for outcome prediction. Selected through L2‐regularized and cross‐validated proportional hazards models, we have developed a novel risk score, identifying patients with low risk for developing metastases even amongst patients with LN‐positive breast cancers.

There is now increasing interest in the role of the tumour micro‐environment in cancer prognosis and, in particular, the role of lymphocytic infiltrates and stromal reactions. TIL composition within the primary invasive tumour has proven to be superior to the classical tumour, node and metastasis (TNM) staging in predicting outcome in some series 12. TIL composition has also been shown to be predictive of response to chemotherapeutic agents 8, 20, 21, 22. Much of this, however, relies on complex multi‐parametric surface phenotyping of TILs from fresh frozen or freshly dissociated tissues. Unsurprisingly, given the technical challenges, financial constraints, and operator dependence, these immune scoring systems have not been widely adopted in routine clinical practice despite their potential benefits. Our work provides a novel scoring system that can accurately predict DMFS, particularly in LN‐positive and TNBCs, based on routine H&E histopathological examination. Hence, unlike complex multi‐parametric assays, our method could be easily integrated into standard clinical practice.

To our knowledge, we are the first to include the histological features of uninvolved LNs into a predictive model of DMFS for breast cancer patients. Although draining LNs are the first site of metastasis for many cancers, the histological progression from an uninvolved to an involved LN remains poorly documented. A recent study in murine models found that the stromal compartments in uninvolved LNs undergo structural reorganisation due to the proliferation and transcriptional changes of fibroblastic reticular cells, potentially providing a pro‐tumour environment 22. While we cannot attribute changes to specific cell types within the uninvolved LNs, we observed in this study that patients with shorter time to any distant metastasis had fewer and larger GCs, which were predominantly located in the centre of the node. GCs are dynamic structures where B cells expressing high‐affinity, potentially tumour‐reactive antibodies mature into antibody‐secreting plasma cells and memory B cells. Further studies are clearly warranted to elucidate molecular signals in uninvolved LNs that may predict development of nodal metastases.

The presence of lymphocytic lobulitis adjacent to the primary tumour was another feature associated with a reduced risk of recurrence in all breast cancers and TNBCs, including LN‐positive cohorts. Lymphocytic lobulitis is characterised by perilobular and perivascular aggregates of B and T lymphocytes with increased expression of MHC class II antigens by the lobular and ductal epithelium and has been described in prophylactic mastectomies from women with *BRCA1*/2 mutations 23. In normal breast tissue, immune cells are predominantly localised to lobules. In lymphocytic lobulitis, disproportionately higher numbers of T cells (CD4 and CD8) and B cells (CD20) are seen when compared with dendritic cells or monocytes/macrophages. The role of these cells in the breast is not entirely clear, although a role in tissue immune surveillance has been proposed 24.

Through incorporating the variability of immune and stromal composition at the primary tumour bed, along with architectural changes in uninvolved and involved LNs, we were able to develop an ISH risk score to identify low‐risk patients among LN‐positive breast cancers. Effective biomarkers to guide clinical management are of particular importance in high‐risk patients such as those with TNBC and LN‐positive disease. These patients are often referred for adjuvant chemotherapy but only a proportion benefit from increased overall survival. Currently, there is a lack of validated biomarkers to identify patients for whom less aggressive intervention might be appropriate. Our ISH risk score was derived through an unbiased approach, in which we aimed to: (1) quantify the outcome prediction performance of regression results; (2) avoid overfitting via monitoring of outcome prediction on unseen data; and (3) construct a multivariate risk signature based on the optimised covariate set. Thus, we propose that our ISH risk score might be used for effective patient stratification, although clearly this requires further evaluation.

This study has several limitations. Firstly, the breast cancer cohort used was enriched for TNBCs (170/309) which frequently have increased immune infiltrates compared with other subtypes of breast cancer. Thus, future studies in cohorts with higher proportions of ER‐positive or HER2‐positive breast cancers are warranted to corroborate the applicability of these histomorphological features. Secondly, as a high proportion of tumours, particularly TNBCs, were of histological grade 3, this pathological characteristic was non‐discriminatory for risk prediction in this cohort. In cohorts with more typical distribution of histological grade of consecutive series of invasive breast cancers, the inclusion of histological grade in the multivariate model will need to be evaluated. Finally, we have not as yet sub‐categorised immune and stromal cells in uninvolved LNs, particularly in the GCs, with orthogonal experiments such as IHC or immunofluorescence techniques. Although this would shed further light on the pro‐tumour evolving micro‐environment in these lymphoid organs, they are beyond the scope of this manuscript.

In conclusion, our study highlights the added value of comprehensive histopathological examination of tumoural, peritumoural, and nodal features for the prediction of distant metastases. By suppressing overfitting via repeated cross‐validation and constructing reproducible multivariate risk signatures, our mathematical approach provides a robust method for survival analysis beyond the commonly used Kaplan‐Meier analyses. Furthermore, these results point towards a novel histopathological prognostic tool that improves 5‐year DMFS prediction accuracy in high‐risk breast cancers and if validated, could be implemented in standard histological practice.

## Author contributions statement

AG, PG, AT, SP: concept and design; PG, AG, JO, CG, AT, SP: data acquisition; AG, PG, TP, ACCC, KN, YW, AT, SP: data analysis and interpretation; AG, PG, TP, SI, RM, YW, AT, ACCC, SP: wrote the manuscript; AG, PG, ACCC, TP, SI, KN, YW, AT, SP: critically reviewed the manuscript.

## Supporting information

SUPPLEMENTARY MATERIAL ONLINE

**Supplementary materials and methods**

**Figure S1.** Smooth muscle actin (SMA) and alcian blue staining of selected primary tumours with differing stromal features.

**Figure S2.** Germinal centres of the LNs stained with CD20 (B cell marker) and CD11c (dendritic cell marker).

**Figure S3.** Identification of overfitting point in the Bayesian batch Cox analysis. Graphs illustrating the fraction of correctly predicted disease outcome for patients (i.e. those who had an event prior the cut‐off time) (prediction time point) over those patients who had either never distant metastasis or developed metastasis after this time point. The top and bottom lines represent the validation and test sets, respectively. The number of covariates used for the prediction is shown on the *x*‐axis (*nr of covs*). Iteratively, covariates are removed from the analysis

**Figure S4.** Correlations analysis of covariates. Plot showing all pairwise Pearson's correlations for standard clinical features and all novel morphological assessed features across 309 breast carcinomas in (A) and for all histopathological characteristics across TNBC (B). The list of covariates is provided at the bottom, whereby immune‐associated features are indicated in blue, features assessed in the uninvolved lymph node in purple, in the involved lymph node in green, and standard clinico‐pathological features in black. We also included the relevant outcome variable (TTE – time to event)

**Figure S5.** Optimised proportional hazards models to identify covariates for the prediction of distant metastasis‐free survival (DMFS) in all breast cancers LN‐negative and TNBC LN‐negative cohorts

**Table S1.** Histopathological evaluation of primary tumour micro‐environment, uninvolved lymph nodes, and involved lymph nodes

**Table S2.** Results from multivariate L2‐regularised and cross‐validated proportional hazard analysis using groups A, B, and C of immune and stroma histomorphologically assessed and clinico‐pathological characteristics. Taken at the 5‐year prediction cut‐off time point
