# Supplementary material for: Histological scoring of immune and stromal features in breast and axillary lymph nodes is prognostic for distant metastasis in lymph node‐positive breast cancers
Source: J Pathol Clin Res. 2018 Jan 8;4(1):39–54. doi: 10.1002/cjp2.87 (PMC5783956; doi:10.1002/cjp2.87)
Supplement: Supplementary file 1 — Supplementary materials and methods [file CJP2-4-39-s006.docx]

Supplementary methods

Statistical survival analysis

Our motivation was to quantify the degree to which the histological and clinicopathological features assessed (the covariates) carried information to predict whether unseen patients would have distant metastasis or not. Given the large number of covariates, we implemented a multivariate proportional hazards model with L2-regularization and repeated cross-validation-based iterative determination of covariates to prevent overfitting [15–17]. For each chosen prediction time point (e.g. 1 year, 2 years, 3 years etc of DMFS) and each set of covariates, patients were randomly dichotomized into groups of equal sizes to serve as training and validation sets. In the training set, the regression parameters were determined on the basis of maximum *a posteriori* Bayesian probability (MAP) with Gaussian parameter priors (equivalent to L2-penalized Cox regression) [18,19]. These parameters were then used in combination with Breslow’s estimator for the base hazard rate to predict clinical outcome for those patients who had not been censored at the chosen time point in both the training and the validation set. Covariates were all normalized to zero average and unit variance so that the Gaussian priors for regression parameters could be given fixed unit widths. Predicted binary outcomes (i.e. relapse most likely before or after the chosen cut-off time point) were compared to the true outcomes (the dotted line in Figure 4) and the quality of prediction, after averaging over the results of 100 repetitions, was reported as the fraction of correctly predicted outcomes in both sets and displayed graphically. Similarly, one can extract estimates of averages and confidence intervals of regression parameters.

This procedure was repeated for different covariate sets, starting with all covariates and then iteratively reducing the number of covariates (via successive removal of the least informative factor at each stage) until all were disregarded (see supplementary material, Figure S3). A ranking of covariates according to their relevance in multivariate regression was thereby determined, without the assumption that the covariates have independent characteristics. The dependence of training and validation prediction performance on the number of retained covariates shows the typical fingerprint of overfitting (see figure below for illustration); a well-defined maximum in the validation performance at a specific optimal point coinciding with the onset of separation of training and validation performance, which defines the optimal set of covariates (e.g. five covariates in graph below). Regression parameters were translated into, and reported as, hazard ratios (HR) and 95% confidence intervals of HR, as stated in the main methods.
