# Supplementary material for: Histological scoring of immune and stromal features in breast and axillary lymph nodes is prognostic for distant metastasis in lymph node‐positive breast cancers
Source: J Pathol Clin Res. 2018 Jan 8;4(1):39–54. doi: 10.1002/cjp2.87 (PMC5783956; doi:10.1002/cjp2.87)
Supplement: Supplementary file 2 — Figure S1. Smooth muscle actin (SMA) and alcian blue staining of selected primary tumours with differing stromal features. [file CJP2-4-39-s001.docx]

**Figure S1.** Smooth muscle actin (SMA) and alcian blue staining of selected primary tumours with differing stromal features.

Exemplars of stromal tissue stained with smooth muscle actin and Alcian blue. (A) Oedematous/myxoid stroma with characteristically vacuolated material admixed with collagen fibres; (B) fibroblastic stroma with numerous stromal cells (fibroblasts); (C) hyalinized stroma. Figures show tissue stained with H&E; SMA (Dako Ab Cat. #0851, antibody used at 1:200 concentration, antigen retrieval performed with 18 min microwaving in citric buffer pH 6.); and Alcian blue staining (Alcian blue 8GX, Generon).

**
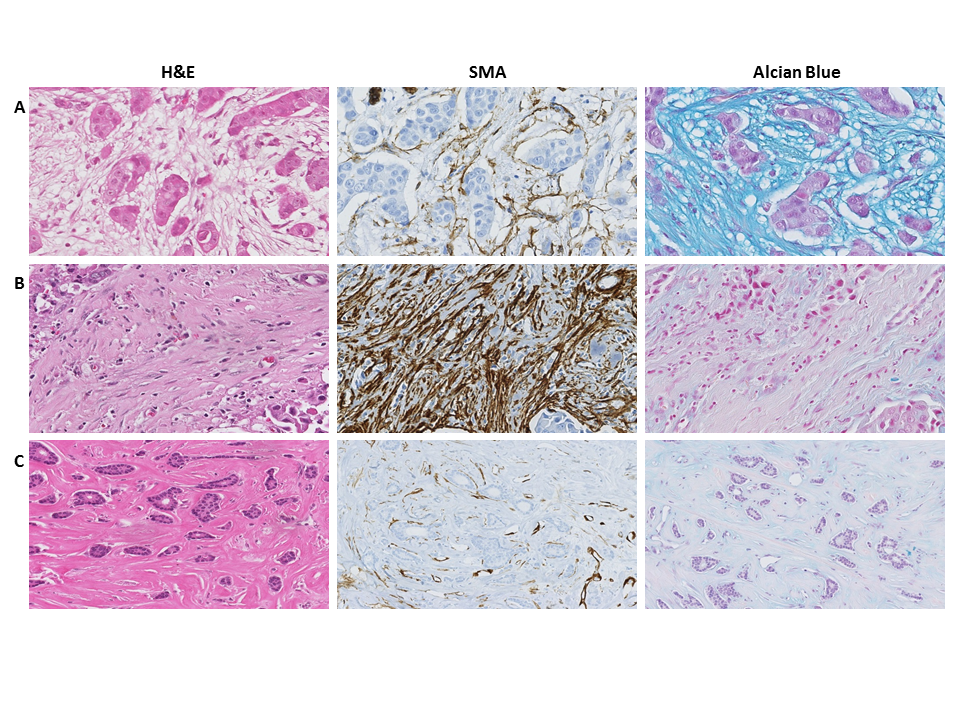
**

Quantification of SMA with HistoQuest. (A) Oedematous/myxoid stroma; (B) fibroblastic stroma; (C) hyalinized stroma.


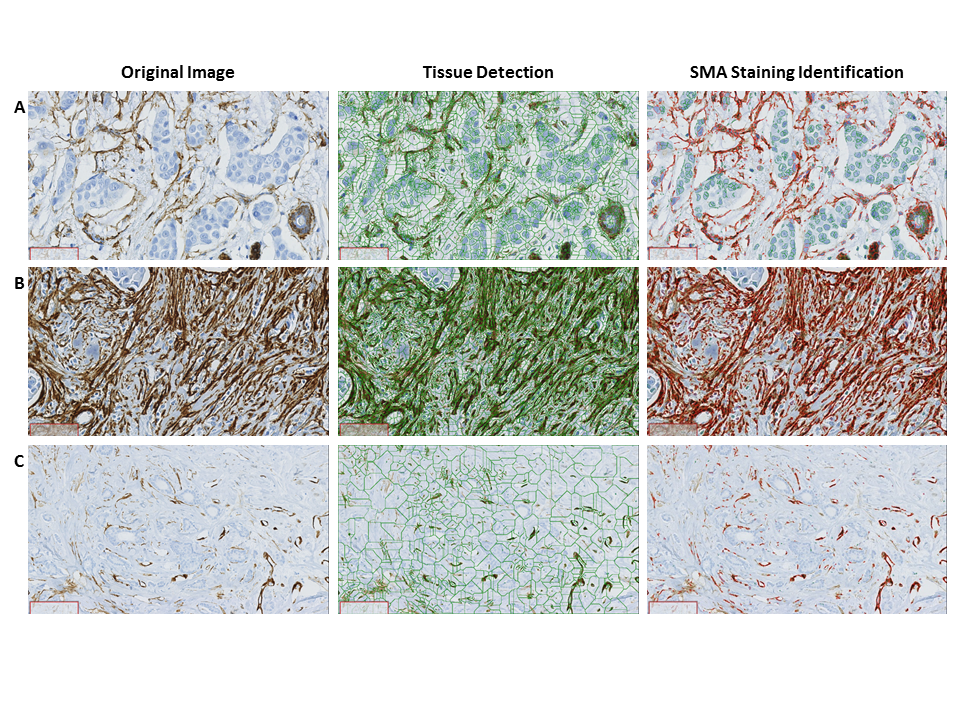


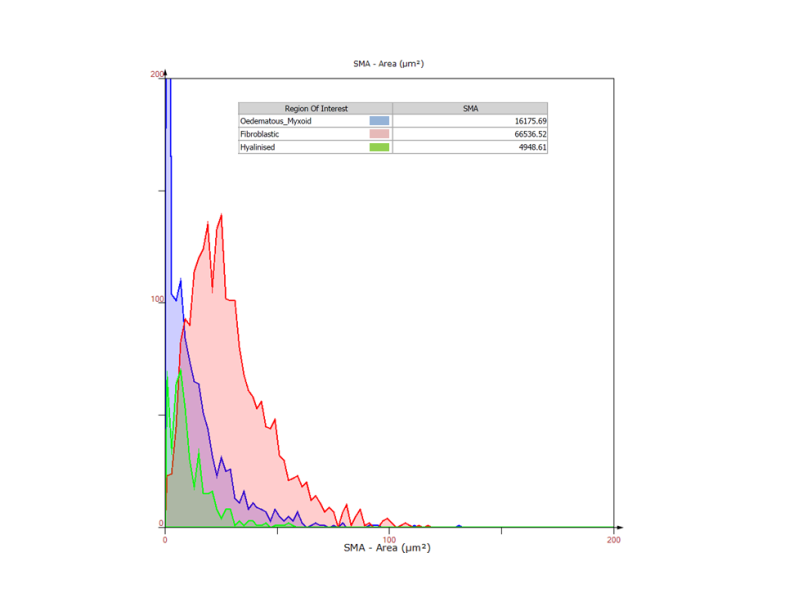


Histograms of DAB stained surface areas in µm^2^.
