# Supplementary material for: Histological scoring of immune and stromal features in breast and axillary lymph nodes is prognostic for distant metastasis in lymph node‐positive breast cancers
Source: J Pathol Clin Res. 2018 Jan 8;4(1):39–54. doi: 10.1002/cjp2.87 (PMC5783956; doi:10.1002/cjp2.87)

**Figure S2.** Germinal centres of the LNs stained with CD20 (B cell marker) and CD11c (dendritic cell marker).

I (A) Lymph node with predominantly primary follicles (H&E); (B) lymph node with predominantly primary follicles (CD20); (C) lymph node with secondary follicles with germinal centres and sinus histiocytosis (H&E); (D) lymph node with secondary follicles with germinal centres (CD20).

**
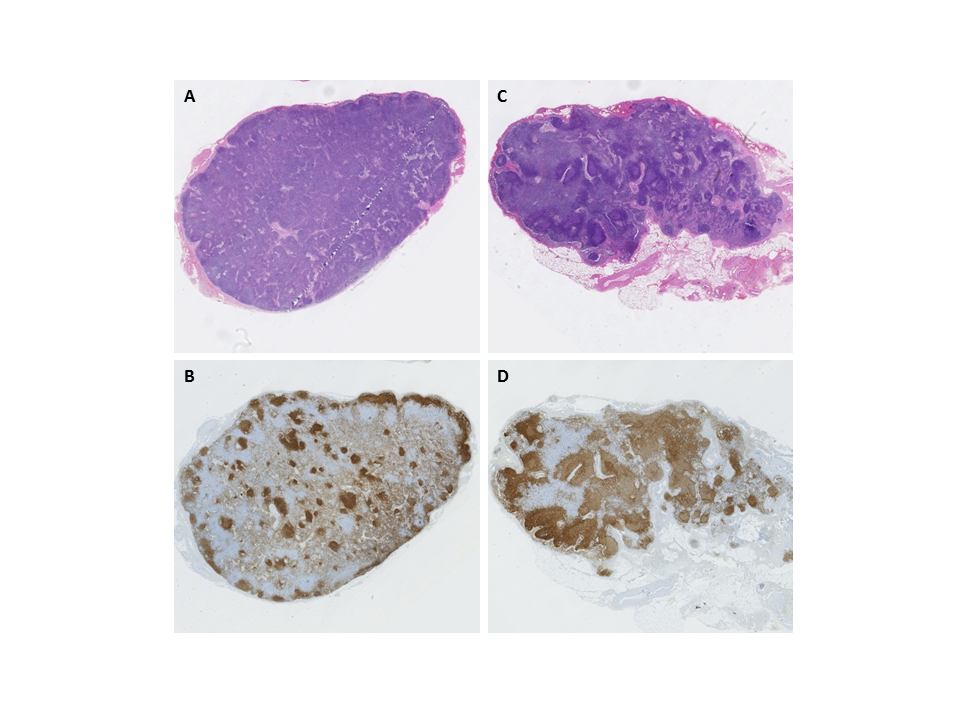
**

II (A) Primary follicles; (B**)** germinal centre; tissue stained with a B cell marker CD20 [Dako Cat. #7019, citrate buffer, 1:50 dilution] and a dendritic cell marker CD11c [Abcam Cat. #52631, citrate buffer, 1:100 dilution].


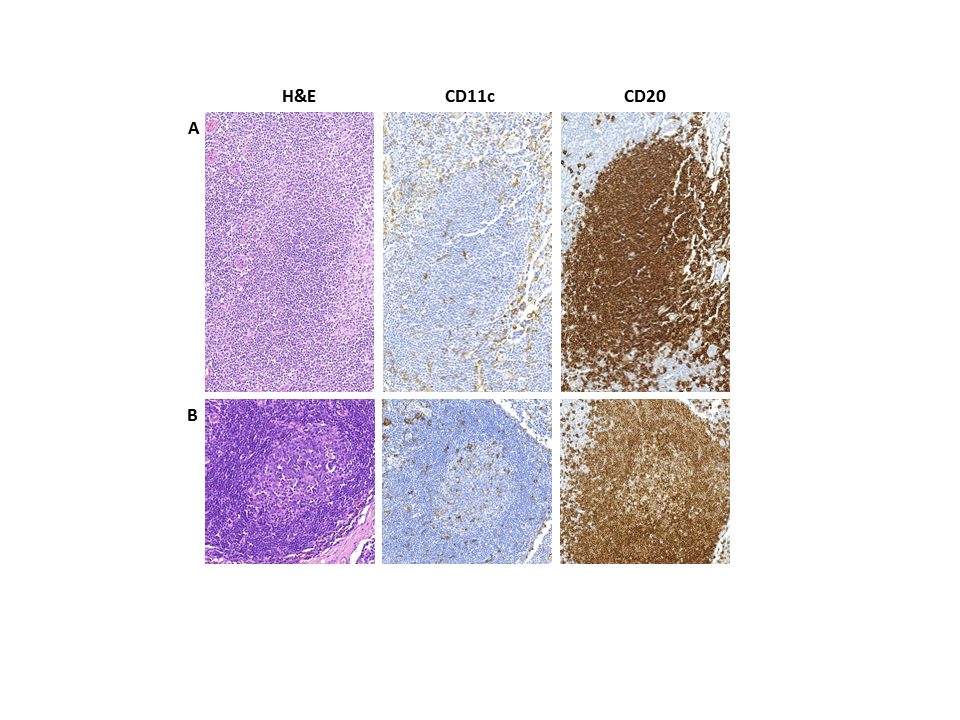

Supplement: Supplementary file 3 — Figure S2. Germinal centres of the LNs stained with CD20 (B cell marker) and CD11c (dendritic cell marker). [file CJP2-4-39-s002.docx]
