# Supplementary material for: Histological scoring of immune and stromal features in breast and axillary lymph nodes is prognostic for distant metastasis in lymph node‐positive breast cancers
Source: J Pathol Clin Res. 2018 Jan 8;4(1):39–54. doi: 10.1002/cjp2.87 (PMC5783956; doi:10.1002/cjp2.87)

Figure S3**.** Identification of overfitting point in the Bayesian batch Cox analysis.

Graphs illustrating the fraction of correctly predicted disease outcome for patients (i.e. those who had an event prior the cut-off time) (prediction time point) over those patients who had either never distant metastasis or developed metastasis after this time point. The top and bottom lines represent the validation and test sets, respectively. The number of covariates used for the prediction is shown on the *x*-axis (*nr of covs*). Iteratively, covariates are removed from the analysis.


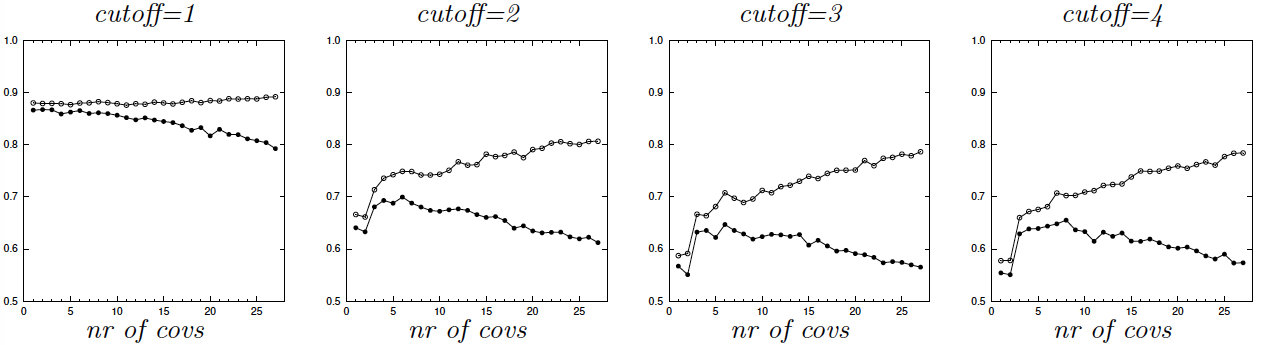

Supplement: Supplementary file 4 — Figure S3. Identification of overfitting point in the Bayesian batch Cox analysis. Graphs illustrating the fraction of correctly predicted disease outcome for patients (i.e. those who had an event prior the cut‐off time) (prediction time point) over those patients who had either never distant metastasis or developed metastasis after this time point. The top and bottom lines represent the validation and test sets, respectively. The number of covariates used for the prediction is shown on the x‐axis (nr of covs). Iteratively, covariates are removed from the analysis [file CJP2-4-39-s003.docx]
