# Supplementary figures and images for: Histological scoring of immune and stromal features in breast and axillary lymph nodes is prognostic for distant metastasis in lymph node‐positive breast cancers
Source: J Pathol Clin Res. 2018 Jan 8;4(1):39–54. doi: 10.1002/cjp2.87 (PMC5783956; doi:10.1002/cjp2.87)

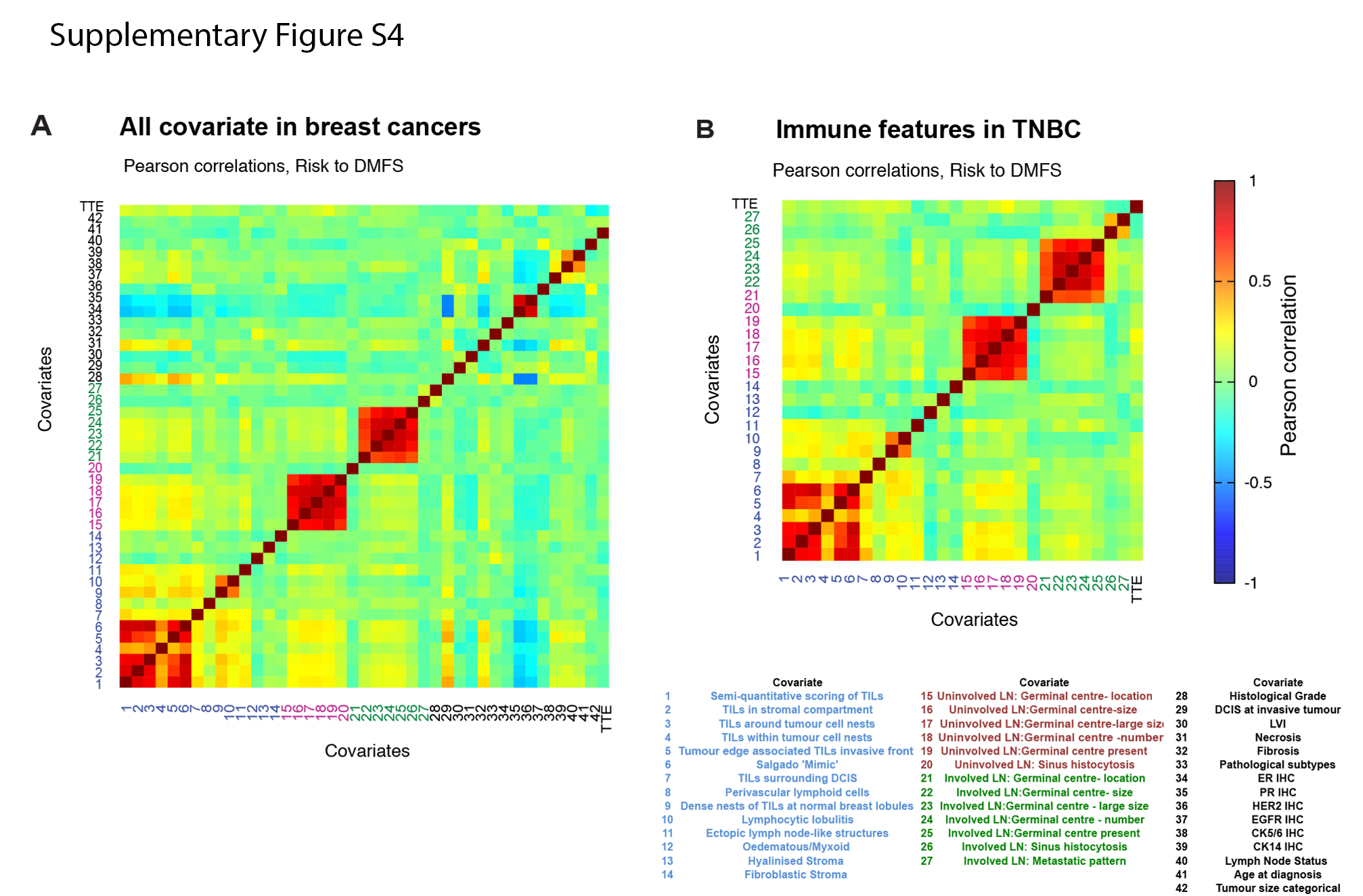

Supplement: Supplementary file 5 — Figure S4. Correlations analysis of covariates. Plot showing all pairwise Pearson's correlations for standard clinical features and all novel morphological assessed features across 309 breast carcinomas in (A) and for all histopathological characteristics across TNBC (B). The list of covariates is provided at the bottom, whereby immune‐associated features are indicated in blue, features assessed in the uninvolved lymph node in purple, in the involved lymph node in green, and standard clinico‐pathological features in black. We also included the relevant outcome variable (TTE – time to event) [file CJP2-4-39-s004.tif]

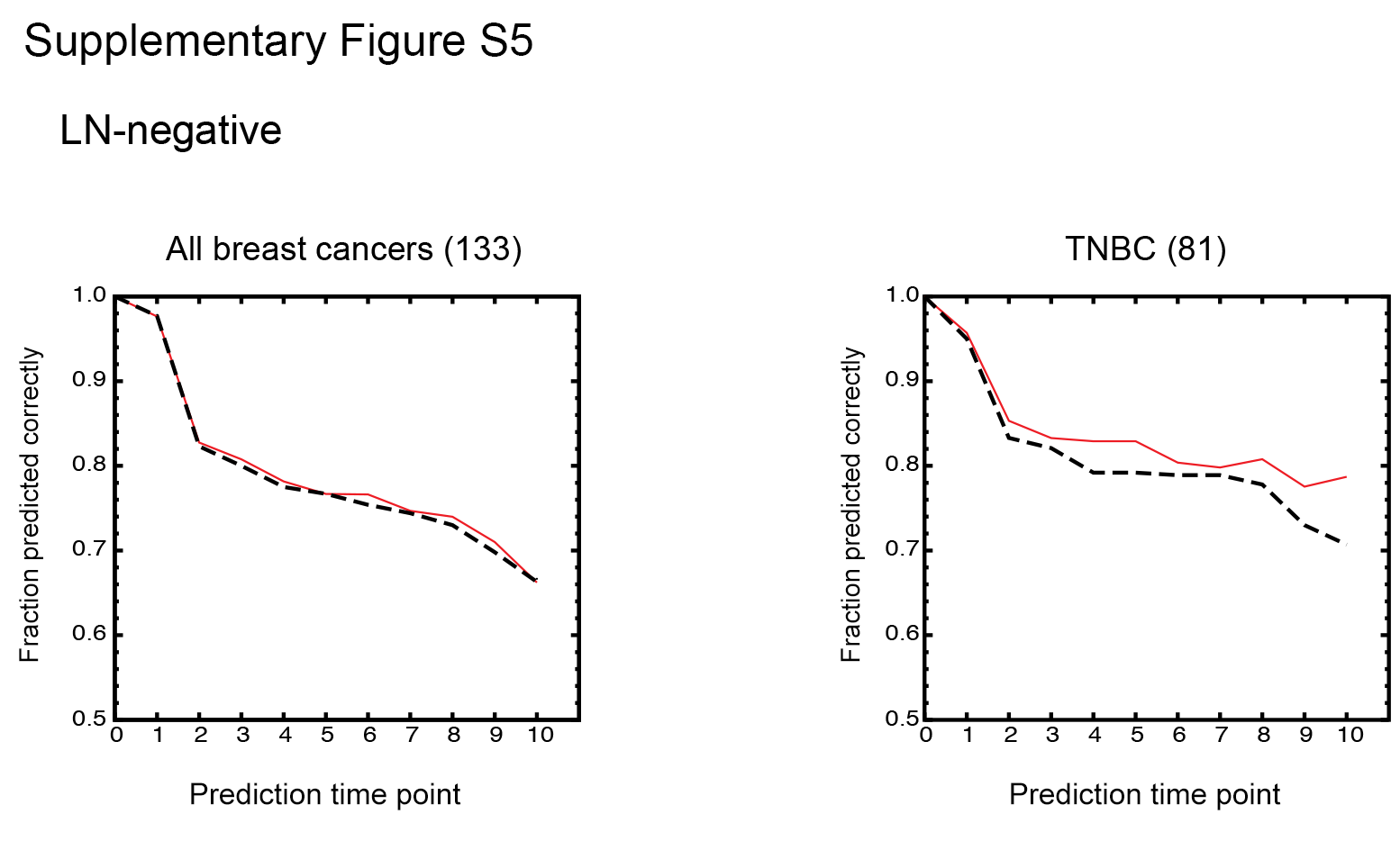

Supplement: Supplementary file 6 — Figure S5. Optimised proportional hazards models to identify covariates for the prediction of distant metastasis‐free survival (DMFS) in all breast cancers LN‐negative and TNBC LN‐negative cohorts [file CJP2-4-39-s005.tif]
