# Supplementary material for: Histological scoring of immune and stromal features in breast and axillary lymph nodes is prognostic for distant metastasis in lymph node‐positive breast cancers
Source: J Pathol Clin Res. 2018 Jan 8;4(1):39–54. doi: 10.1002/cjp2.87 (PMC5783956; doi:10.1002/cjp2.87)
Supplement: Supplementary file 7 — Table S1. Histopathological evaluation of primary tumour micro‐environment, uninvolved lymph nodes, and involved lymph nodes [file CJP2-4-39-s007.docx]

Table S1. Histopathological evaluation of primary tumour micro-environment, uninvolved lymph nodes, and involved lymph nodes

(A) Primary tumour

| Intratumoural | Score | Score legend | Peritumoural | Score | Score legend | Stromal features | Score |
| --- | --- | --- | --- | --- | --- | --- | --- |
| Semi-quantitative assessment of lymphocytic infiltration across entire tumour | 0–4 | 0: Absence of lymphocytes  1: Minimal  (1 to <10%)  2: Mild  (10–20%)  3: Moderate  (>20 to <50%)  4: Strong (≥50%) | Lymphoid infiltrate surrounding DCIS | 0–3 | 0: Absence of lymphocytes  1: Mild  (10–20%)  2: Moderate (>20 to <50%)  3: Strong (≥50%) | Oedematous/Myxoid stroma | Absent  Present  Dominant |
| TILs scattered in the intratumoural stroma | 0–4 |  | Lymphocytes surrounding normal breast lobules | 0–3 |  |  |  |
|  |  |  |  |  |  | Hyalinized stroma |  |
| TILs around tumour cell nests | 0–4 |  |  |  |  |  |  |
|  |  |  | Perivascular lymphoid infiltrate | 0–3 |  |  |  |
| TILs at the invasive margin | 0–4 |  |  |  |  |  |  |
|  |  |  | Lymphocytic lobulitis | Present/Absent |  | Fibroblastic stroma |  |
| Intratumoural TILs | Present/Absent |  |  |  |  |  |  |
|  |  |  | Tertiary lymphoid structures |  |  |  |  |
| Salgado’s classification | 0–10% stromal TILs  20–40% stromal TILs  50–90% stromal TILs | |  | | | | |

(B) Lymph nodes

| Feature | Score/Assessment |
| --- | --- |
| Germinal centres: numbers Germinal centre: location  Germinal centre: size | No // Few // Moderate // Marked  Peripheral // Predominantly Peripheral // Central // Predominantly Central // Mixed (both Peripheral and Central)  Small // Moderate // Large // Mixed |
| Sinus histiocytosis | grade 0 (absent),  grade 1 (<2 cells),  grade 2 (2–4 cells),  grade 3 (>4 to <8 cells)  grade 4 (≥8 cells across the sinus) |
| Pattern of metastasis | Sub-capsular // Intrasinusoidal // Diffuse // Nodular// Mixed// Total metastatic replacement of LN |
