# Supplementary material for: Histological scoring of immune and stromal features in breast and axillary lymph nodes is prognostic for distant metastasis in lymph node‐positive breast cancers
Source: J Pathol Clin Res. 2018 Jan 8;4(1):39–54. doi: 10.1002/cjp2.87 (PMC5783956; doi:10.1002/cjp2.87)
Supplement: Supplementary file 8 — Table S2. Results from multivariate L2‐regularised and cross‐validated proportional hazard analysis using groups A, B, and C of immune and stroma histomorphologically assessed and clinico‐pathological characteristics. Taken at the 5‐year prediction cut‐off time point [file CJP2-4-39-s008.docx]

**Table S2.** Results from multivariate Bayesian Cox regression analysis using groups A, B, and C of immune histomorphological and clinicopathological characteristics

Taken at the 5-year prediction cut-off time point.

(A) All breast cancers (309) – all features (42 covariates)

| Covariate | Multivariate Cox | Multivariate HR | Association to shorter DMFS |
| --- | --- | --- | --- |
| LN-status | 0.405 ± 0.111 | 2.248 | LN positive |
| ER-status | −0.178 ± 0.133 | 0.7 | ER negative |
| HER2-status | 0.285 ± 0.101 | 1.768 | HER2 positive |
| Fibrosis | 0.221 ± 0.098 | 1.555 | Present within the tumour or in peritumoural areas |
| Salgado’s classification | −0.232 ± 0.129 | 0.629 | 0–10% stromal TILs |
| TILs at the invasive margin | −0.338 ± 0.146 | 0.509 | Minimal (1 to <10%) |
| Germinal centre semi-quantitative assessment in uninvolved LN | −0.698 ± 0.222 | 0.248 | Few |
| Germinal centre size in uninvolved LN | 0.364 ± 0.181 | 2.071 | Large size |
| Germinal centre hyperplasia in uninvolved LN | 0.232 ± 0.179 | 1.59 | Present |
| Germinal centre location in involved LN | 0.556 ± 0.185 | 3.04 | Predominantly in the centre |
| Germinal centre semi-quantitative assessment in involved LN | −0.212 ± 0.231 | 0.654 | Few |

All breast cancers (309) – immune features (25 covariates):

| Covariates | Multivariate Cox | Multivariate HR | Association to shorter DMFS |
| --- | --- | --- | --- |
| Salgado criteria | −0.200 ± 0.146 | 0.67 | 0–10% stromal TILs |
| Germinal centre semi-quantitative assessment in uninvolved LN | −0.527 ± 0.157 | 0.348 | Few |
| Germinal centre size in uninvolved LN | 0.398 ± 0.149 | 2.216 | Large size |
| Germinal centre location in involved LN | 0.538 ± 0.189 | 2.932 | Predominantly in the centre |
| Germinal centre semi-quantitative assessment in involved LN | −0.307 ± 0.206 | 0.541 | Few |

All breast cancers (309) – standard features (8 covariates):

| Covariates | Multivariate Cox | Multivariate HR | Association to shorter DMFS |
| --- | --- | --- | --- |
| LN-status | 0.322 ± 0.091 | 1.904 | LN positive |
| HER2-status | 0.293 ± 0.085 | 1.797 | HER2 positive |
| LVI-status | 0.197 ± 0.082 | 1.483 | LVI present |

TNBC (170 )– all features (42 covariates):

| Covariate | Multivariate Cox | Multivariate HR | Association to shorter DMFS |
| --- | --- | --- | --- |
| LN-status | 0.246 ± 0.176 | 1.635 | LN positive |
| TILs at the invasive margin | −0.28 ± 0.156 | 0.571 | Minimal (1 to <10%) |
| Lymphocytic lobulitis | −0.415 ± 0.428 | 0.436 | Absent |
| Lymphoid infiltrate surrounding DCIS | −0.513 ± 0.218 | 0.358 | Absent |
| Tertiary lymphoid structures | 0.416 ± 0.158 | 2.298 | Present |
| Oedematous/Myxoid stroma | 0.318 ± 0.161 | 1.889 | Present |
| Germinal centre location in involved LN | 0.662 ± 0.324 | 3.758 | Predominantly in the centre |
| Germinal centre semi-quantitative assessment in uninvolved LN | −0.398 ± 0.148 | 0.451 | Few |
| Germinal centre hyperplasia in involved LN | −0.64 ± 0.444 | 0.278 | Absent |
| Metastatic pattern in involved LN | 0.707 ± 0.252 | 4.11 | Mixed |
| Germinal centre size in involved LN | 0.627 ± 0.423 | 3.504 | Large size |
| Germinal centre semi-quantitative assessment in involved LN | −0.351 ± 0.502 | 0.496 | Few |

TNBC (170) – immune features (25 covariates):

| Covariates | Multivariate Cox | Multivariate HR | Association to shorter DMFS |
| --- | --- | --- | --- |
| Lymphocytic lobulitis | −0.499 ± 0.303 | 0.368 | Absent |
| Germinal centre semi-quantitative assessment in uninvolved LN | −0.441 ± 0.148 | 0.413 | Few |
| Germinal centre location in involved LN | 0.613 ± 0.282 | 3.401 | Predominantly in the centre |
| Germinal centre hyperplasia in involved LN | −0.496 ± 0.268 | 0.371 | Absent |
| Metastatic pattern in involved LN | 0.399 ± 0.218 | 2.221 | Mixed |
|  |  |  |  |

TNBC (170) – standard features (8 covariates):

| Covariates | Multivariate Cox | Multivariate HR | Association to shorter DMFS |
| --- | --- | --- | --- |
| LVI-status | 0.248 ± 0.134 | 1.642 | LVI present |
| LN-status | 0.221 ± 0.135 | 1.558 | LN positive |

(B) LN-positive patients

LN-positive all breast cancers (143) – immune features (25 covariates):

| Covariates | Multivariate Cox | Multivariate HR | Association to shorter DMFS |
| --- | --- | --- | --- |
| Lymphocytic lobulitis** | −0.263 ± 0.228 | 0.591 | Absent |
| Salgado classification** | −0.188 ± 0.199 | 0.687 | 0–10% stromal TILs |
| TILs at the invasive margin | −0.359 ± 0.155 | 0.487 | Minimal (1 to <10%) |
| Germinal centre semi-quantitative assessment in uninvolved LN** | −0.774 ± 0.229 | 0.212 | Few |
| Germinal centre size in uninvolved LN** | 0.489 ± 0.197 | 2.659 | Large size |
| Germinal centre location in involved LN** | 0.402 ± 0.178 | 2.234 | Predominantly in the centre |

**Used for ImmunoHisto Risk Score.

LN-positive TNBC (64) – immune features (25 covariates):

| Covariates | Multivariate Cox | Multivariate HR | Association to shorter DMFS |
| --- | --- | --- | --- |
| Salgado criteria** | −0.633 ± 0.332 | 0.282 | 0–10% stromal TILs |
| Lymphocytic lobulitis** | −0.54 ± 0.258 | 0.339 | No normal breast lobules |
| Germinal centre semi-quantitative assessment in uninvolved LN** | −1.103 ± 0.312 | 0.11 | Few |
| Germinal centre size in uninvolved LN** | 0.499 ± 0.319 | 2.713 | Large size |
| Germinal centre location in involved LN** | 0.532 ± 0.296 | 2.898 | Predominantly in the centre |
| Germinal centre hyperplasia in involved LN | −0.203 ± 0.622 | 0.666 | Absent |
| Metastatic pattern in involved LN | 0.602 ± 0.234 | 3.334 | Mixed |

**Used for ImmunoHisto Risk Score

(C) LN-negative cancers patients

LN-negative all breast cancers (133) – immune features (25 covariates):

| Covariates | Multivariate Cox | Multivariate HR | Association to shorter DMFS |
| --- | --- | --- | --- |
| Oedemamtous/Myxoid stroma | 0.4 ± 0.185 | 2.225 | Present |

LN-negative TNBC (81) – immune features (25 covariates):

| Covariates | Multivariate Cox | Multivariate HR | Association to shorter DMFS |
| --- | --- | --- | --- |
| Lymphocytic lobulitis | −0.85 ± 0.161 | 0.196 | Absent |
| Oedematous/Myxoid stroma | 0.69 ± 0.265 | 3.32 | Present |
| Tertiary lymphoid structures | 0.403 ± 0.209 | 2.24 | Present |
| Germinal centre semi-quantitative assessment in involved LN | −0.426 ± 0.277 | 0.426 | Few |
